# Host Intrinsic and Innate Intracellular Immunity During Herpes Simplex Virus Type 1 (HSV-1) Infection

**DOI:** 10.3389/fmicb.2019.02611

**Published:** 2019-11-08

**Authors:** Thamir Alandijany

**Affiliations:** ^1^Department of Medical Laboratory Technology, Faculty of Applied Medical Sciences, King Abdulaziz University, Jeddah, Saudi Arabia; ^2^Special Infectious Agents Unit, King Fahd Medical Research Center, King Abdulaziz University, Jeddah, Saudi Arabia

**Keywords:** intracellular immunity, innate, intrinsic, HSV-1, ICP0, antiviral, interferons, PML-NBs

## Abstract

When host cells are invaded by viruses, they deploy multifaceted intracellular defense mechanisms to control infections and limit the damage they may cause. Host intracellular antiviral immunity can be classified into two main branches: (i) intrinsic immunity, an interferon (IFN)-independent antiviral response mediated by constitutively expressed cellular proteins (so-called intrinsic host restriction factors); and (ii) innate immunity, an IFN-dependent antiviral response conferred by IFN-stimulated gene (ISG) products, which are (as indicated by their name) upregulated in response to IFN secretion following the recognition of pathogen-associated molecular patterns (PAMPs) by host pattern recognition receptors (PRRs). Recent evidence has demonstrated temporal regulation and specific viral requirements for the induction of these two arms of immunity during herpes simplex virus type 1 (HSV-1) infection. Moreover, they exert differential antiviral effects to control viral replication. Although they are distinct from one another, the words “intrinsic” and “innate” have been interchangeably and/or simultaneously used in the field of virology. Hence, the aims of this review are to (1) elucidate the current knowledge about host intrinsic and innate immunity during HSV-1 infection, (2) clarify the recent advances in the understanding of their regulation and address the distinctions between them with respect to their induction requirements and effects on viral infection, and (3) highlight the key roles of the viral E3 ubiquitin ligase ICP0 in counteracting both aspects of immunity. This review emphasizes that intrinsic and innate immunity are temporally and functionally distinct arms of host intracellular immunity during HSV-1 infection; the findings are likely pertinent to other clinically important viral infections.

## Introduction

Intracellular immunity represents the front line of host defense against herpes simplex virus type 1 (HSV-1) infection, as for other invading pathogens. HSV-1 is a highly contagious virus that infects approximately 3.7 billion people under the age of 50 worldwide (Looker et al., 2015). It is mainly transmitted via direct contact with infected individuals but the virus can also pass from infected pregnant mothers to their infants (Kriebs, 2008; Looker et al., 2017). The infection is usually asymptomatic or associated with mild symptoms (e.g., cold sores). However, it can lead to serious or even life-threatening outcomes (e.g., keratitis and encephalitis) in neonates and immunocompromised individuals (Simmons, 2002; Herget et al., 2005; Whitley and Baines, 2018). Epithelial cells are the primary sites for lytic replication. The virus is then transported to the trigeminal ganglia of infected hosts, where it establishes a lifelong latent infection. Periodic viral reactivation causes episodes of recurrent disease with variable severity, and this allows transmission to new hosts (Grinde, 2013). Due to its key role in determining the outcomes of infection, the molecular basis of host intracellular immunity during HSV-1 infection has been extensively studied. The current knowledge in the field enables this multifaceted system to be divided into two distinct branches: (i) intrinsic and (ii) innate immunity. However, by carefully reading the literature, these two terms (intrinsic and innate) have been found to be interchangeably and/or simultaneously used in many instances. Hence, the main aim of this review is to highlight the distinction and summarize the differences between intrinsic and innate immunity. The nature, orchestration, induction requirements, antiviral effects, and viral counteraction of these two arms of immunity are discussed. To delve into these concepts, it is important to initially start with a brief overview of the virion structure and replication cycle.

## Virion Structure

**FIGURE 1:**
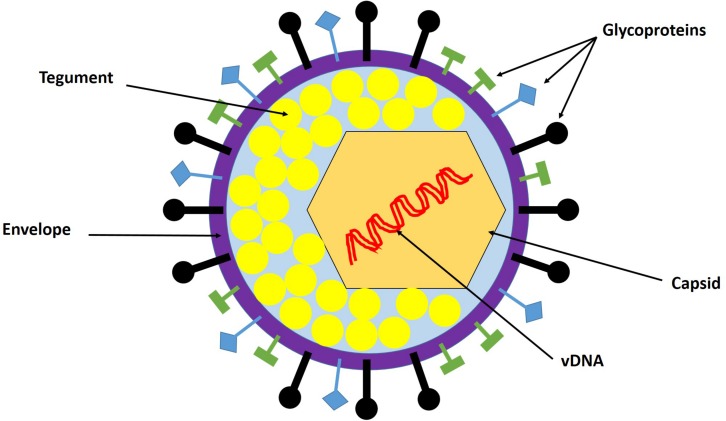
HSV-1 virion structure. The virion is composed of the viral genome, capsid, tegument, and envelope. The viral genome is a linear double-stranded DNA (dsDNA) enclosed in the capsid. The tegument is the protein matrix between the capsid and the envelope. The envelope is a lipid bilayer membrane with glycoprotein projections embedded in it. Adapted with permission from Alandijany (2018).

The HSV-1 virion is a spherical particle with an average diameter of 186 nm (Grunewald et al., 2003). It comprises four components: the core, capsid, tegument, and envelope (Figure 1). The core contains a linear double-stranded DNA (dsDNA) genome packaged as a toroid or spool (Furlong et al., 1972; Zhou et al., 1999). However, in the absence of protein synthesis, this linear DNA is circularized rapidly after nuclear entry (Poffenberger and Roizman, 1985). Complete genome sequencing revealed that the HSV-1 genome is approximately 152 kb in size, comprises 68.3% guanine and cytosine, and exhibits little variation among strains. The viral genome consists of two elements: unique long (UL) and unique short (US) regions bracketed by inverted repeats ab and b′a′, and ac and c′a′, respectively (Wadsworth et al., 1975). The core is surrounded by an icosahedral capsid composed of 162 capsomers (Schrag et al., 1989). The polyamines spermidine and spermine in the core neutralize the negative charge on the viral DNA (vDNA), which allows proper folding of the vDNA within the capsid (Gibson and Roizman, 1971). The protein matrix between the outer surface of the capsid and the undersurface of the envelope is called the tegument. It is highly unstructured and comprises more than 20 viral proteins that have been identified by biochemical assays and proteomics analysis (Roller and Roizman, 1992; Zhou et al., 1999; Loret et al., 2008). Tegument proteins regulate many aspects of viral infection, including entry into target cells, nuclear delivery of the viral genome, regulation of viral gene expression, assembly and egress of progeny virions, and host immune evasion (Kelly et al., 2009). The tegument is enclosed in the viral envelope, which consists of a lipid bilayer derived from host cells, with spike-like glycoprotein projections embedded in it (Spear and Roizman, 1967). Thirteen glycosylated envelope proteins (gB–E, and gG–N) and at least two non-glycosylated envelope proteins (UL20 and Us9) have been identified, and they are particularly important for HSV-1 attachment to target cells (Loret et al., 2008).

## Viral Lytic Replication Cycle

**FIGURE 2:**
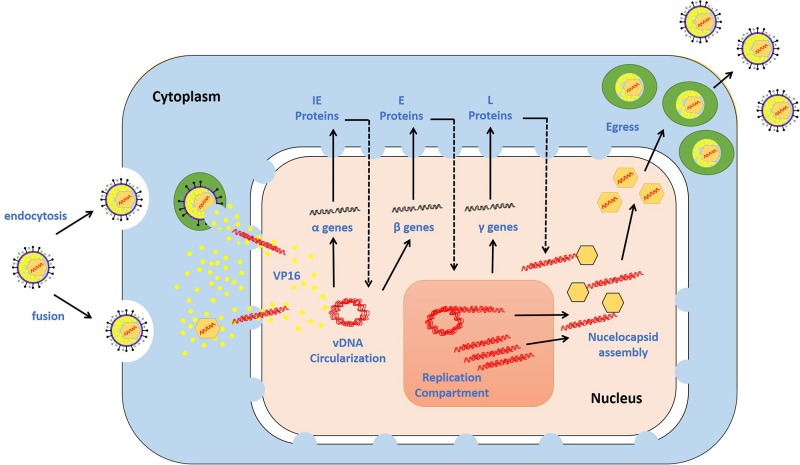
HSV-1 replication cycle. The virus attaches via glycoproteins to cellular receptors. It enters the cells via the fusion of the viral envelope with the plasma membrane or endocytosis. The de-enveloped nucleocapsid is transported to the nuclear pores, and the viral DNA (vDNA) is ejected into the nucleus. The viral genes are transcribed in a temporal cascade: immediate early (IE), early (E), and late (L) proteins. IE protein expression is turned on by the virion-associated protein VP16. E proteins require IE protein synthesis for their expression and play critical roles in triggering vDNA replication. The hypothesized mechanisms underlying vDNA replication involve theta replication followed by rolling circle replication. L protein expression is dependent on vDNA replication. The capsid is assembled at sites adjacent to vDNA replication compartments, permitting the insertion of vDNA into the capsid. The nucleocapsid buds through the nuclear membrane, is transported through the cytoplasm, and fuses with the plasma membrane. During this journey, the nucleocapsid acquires tegument and envelope proteins. The release of mature progeny virions promotes attachment to new cells, and the cycle continues. Adapted with permission from Alandijany (2018).

Epithelial cells represent the primary sites of HSV-1 lytic replication. The replication cycle is initiated when HSV-1 attaches to target cells via interactions between viral glycoproteins and cellular receptors (e.g., heparan sulfate glycosaminoglycans, nectin, herpesvirus entry mediator, and 3-O-sulfated heparin sulfate) (Spear et al., 1992; Geraghty et al., 1998; Warner et al., 1998; Shukla et al., 1999). These interactions enable fusion of the viral envelope with the cellular plasma membrane, and they thereby allow viral entry (Avitabile et al., 2007; Satoh et al., 2008; Gianni et al., 2009). HSV-1 also utilizes endocytosis to enter into some cell types (Nicola, 2016). In the cytoplasm of infected cells, the nucleocapsid is transported to a nuclear pore through the microtubular network. The vDNA remains encapsidated until it is released through a nuclear pore into the nucleus, where it initiates temporally regulated transcription/translation processes, leading to the production of viral immediate early (IE), early (E), and late (L) proteins (Miyamoto and Morgan, 1971; Honess and Roizman, 1974; Kristensson et al., 1986; Sodeik et al., 1997; Wolfstein et al., 2006; Radtke et al., 2010). IE protein (ICP0, ICP4, ICP22, ICP27, and ICP47) expression is mediated by a virion-associated tegument protein, VP16, and cellular factors [e.g., host cell factor 1 (HCF-1) and octamer-binding protein 1 (Oct-1)] (Triezenberg et al., 1988; Stern et al., 1989; Wysocka and Herr, 2003). *De novo* synthesis of IE proteins promotes the expression of E viral genes, which collectively provide the necessary components for triggering vDNA replication. Seven viral gene products have been shown to be essential for vDNA replication: the origin-binding protein UL9, vDNA polymerase catalytic subunit UL30 and its processivity factor UL42, the multifunctional single-stranded (ss) DNA-binding protein ICP8, and the helicase–primase complex (UL5, UL8, and UL52). vDNA replication starts with theta replication and then switches to a rolling circle mechanism, generating long concatemers (Rabkin and Hanlon, 1990; Wilkinson and Weller, 2003). vDNA replication in cooperation with IE proteins stimulates the expression of L proteins (e.g., capsid proteins VP5, VP21, VP23, VP24, and VP26), which enables nucleocapsid assembly and vDNA packaging. The long concatemers are cleaved into unit-length monomers and packaged into capsids (Heming et al., 2017). Newly synthesized nucleocapsids acquire their tegument and some glycoproteins during primary and secondary envelopment, enabling the release of mature infectious progeny virions (Skepper et al., 2001; Owen et al., 2015). An overview of the HSV-1 replication cycle is shown in Figure 2.

## Host Intracellular Immunity

During HSV-1 infection, intracellular immunity plays a central role in determining the fate of incoming virions and, therefore, the consequences of infection (Komatsu et al., 2016). Based on the nature of the effector proteins, induction requirements, and effects on viral replication, host intracellular immunity can be broadly divided into (i) intrinsic and (ii) innate immunity.

### Intrinsic Immunity

Intrinsic immunity is mediated by constitutively expressed host cell restriction factors that can directly and immediately act to control the viral gene expression. The hallmarks of this arm of immunity include the high likelihood of being counteracted by viral proteins, cell specificity, and the potential to be saturated under high multiplicity of infection (MOI) conditions in the absence of viral countermeasures (Bieniasz, 2004; Yan and Chen, 2012; Boutell and Everett, 2013).

A breakthrough in studying intrinsic immunity during HSV-1 infection was the use of an HSV-1 mutant with a null mutation in the viral E3 ubiquitin ligase ICP0 (ΔICP0), which grows poorly under low MOI conditions in some cell types (Stow and Stow, 1986; Sacks and Schaffer, 1987). Indeed, compared with wild-type (WT) virus, ΔICP0 HSV-1 demonstrates a severe replication defect in fibroblast and keratinocytes (∼1000 fold). This replication defect is moderate in cells such as BHK and Vero cells (30–100 fold), while it is almost absent in U2OS and SAOS cells (in which it replicates as efficiently as WT virus). Cells are described as restrictive, semi-permissive, and permissive based on their ability to intrinsically restrict ΔICP0 HSV-1 replication (Yao and Schaffer, 1995; Everett et al., 2004a). Historically, permissive cell lines have been utilized to accurately determine the viral titer of both ΔICP0 and WT HSV-1 stocks, while restrictive cells have been used to investigate host immunity to HSV-1 infection. Importantly, the intrinsic antiviral response to ΔICP0 HSV-1 becomes saturated and no longer effective at increased MOI conditions (Everett et al., 2004a, 2013). A study conducted on human fibroblasts demonstrated that, under low MOI conditions (0.2–1 plaque forming unit (PFU)/cell based on the viral titer in U2OS), ΔICP0 HSV-1 was able to initiate plaque formation only in a minor proportion of infected cells, while the viral genomes remained quiescent in the majority of cells. Correspondingly, at equivalent genome input levels, the gene expression of ΔICP0 HSV-1 was severely restricted in comparison to the gene expression of WT virus. However, the restriction of ΔICP0 HSV-1 replication was relieved under higher MOI conditions (5–10 PFU/cell), leading to a level of replication that was similar to WT virus replication (Everett et al., 2004a). Combined, these studies demonstrated that intrinsic immunity renders some cell types restrictive to HSV-1 infection under low MOI conditions and in the absence of ICP0, which acts as a viral countermeasure.

**FIGURE 3:**
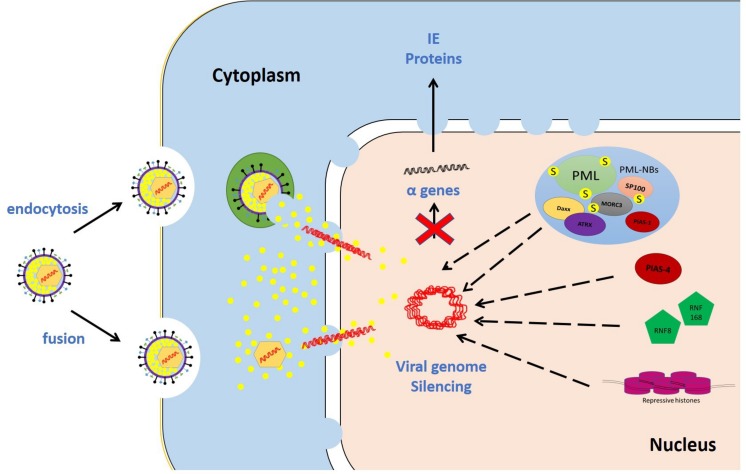
Examples of intrinsic restriction factors that combat HSV-1 infection. As soon as the viral genomes are delivered to the nucleus, intrinsic restriction factors are rapidly recruited to the incoming viral genomes. In the absence of viral countermeasures and under low multiplicity of infection (MOI) conditions, these restriction factor recruitment events induce viral genome silencing. Promyelocytic leukemia protein-nuclear body (PML-NB) constituent proteins (e.g., promyelocytic leukemia, PML; speckled 100 kDa, Sp100; death domain associated protein, Daxx; alpha thalassemia/mental retardation syndrome X-linked, ATRX; and MORC family CW-type zinc finger 3, MORC3), E3 SUMO ligases (e.g., protein inhibitor of activated STAT 1 and 4; PIAS1 and PIAS4, respectively), DNA repair proteins (e.g., ring finger protein-8 and -168; RNF8 and RNF168, respectively), and repressive histones are examples of host intrinsic restriction factors.

Numerous studies were conducted on restrictive cell types to identify intrinsic restriction factors. Examples of these include promyelocytic leukemia protein-nuclear body (PML-NB) constituent proteins (e.g., promyelocytic leukemia, PML; speckled 100 kDa, Sp100; death domain associated protein, Daxx; alpha thalassemia/mental retardation syndrome X-linked, ATRX; and MORC family CW-type zinc finger 3, MORC3), E3 SUMO ligases [e.g., protein inhibitor of activated STAT (PIAS) 1 and 4; PIAS1 and PIAS4, respectively], DNA repair proteins (e.g., ring finger protein-8 and -168; RNF8 and RNF168, respectively), and epigenetic regulators (e.g., repressive histones) (Figure 3).

#### PML-NB Constituent Proteins

PML-NBs, as indicated by their name, are highly dynamic bodies found in the nuclei at about 1–30 PML-NBs per nucleus. They comprise over 70 permanent resident proteins in addition to many transient proteins actively associating and dissociating from these bodies. PML-NB constituent proteins (e.g., PML, SP100, Daxx, ATRX, and MORC3) are involved in the regulation of many cellular processes, including the cell cycle, DNA damage response (DDR), DNA repair, apoptosis, and metabolism (Hsu and Kao, 2018).

Over two decades ago, a disappearance of PML-NBs following HSV-1 infection was observed, which was linked to the expression of the viral IE protein ICP0 (Maul and Everett, 1994). Fluorescence *in situ* hybridization (FISH) experiments demonstrated that, upon nuclear entry, infecting HSV-1 genomes localize at or adjacent to PML-NBs (Maul et al., 1996). This localization was prominent during the initial stage of ΔICP0 HSV-1 infection of cells at the edge of developing plaques (Everett et al., 2004b). In these infected cells, rapid recruitment of PML-NB constituent proteins to dot-like complexes of the vDNA-binding protein ICP4 were observed, with an asymmetric distribution of PML-NB puncta that is distinct from that observed in non-infected cells (Everett et al., 2004b). These viral-induced complexes were shown to contain incoming viral genomes (Everett and Murray, 2005). It was initially unclear whether this phenotype reflected a beneficial or detrimental effect on viral infection. However, accumulating evidence has now conclusively revealed that multiple PML-NB constituent proteins act as host intrinsic restriction factors that restrict ΔICP0 HSV-1 infection.

Utilizing short hairpin RNA technology, individual knockdown of the PML-NB proteins PML, SP100, Daxx, ATRX, or MORC3 clearly enhanced the plaque-forming efficiency and viral protein expression of ΔICP0 HSV-1, but not WT HSV-1 (Everett et al., 2006, 2008a; Lukashchuk and Everett, 2010; Sloan et al., 2016). Although some of these PML-NB proteins are known to be upregulated in response to interferon (IFN), these proteins were found to mediate intrinsic viral restriction independently of IFN production and signaling (Everett et al., 2008b). Moreover, double and triple depletion of PML, SP100, and Daxx additively enhanced the plaque formation of ΔICP0 HSV-1, demonstrating cooperative action to restrict viral replication (Glass and Everett, 2013). However, this additive effect was not sufficient to fully rescue the plaque-formation efficiency of ΔICP0 HSV-1 to the WT virus level, indicating the presence of additional intrinsic restriction factors.

The mechanism underlying PML recruitment to infecting viral genomes is dependent on the SUMO pathway (Cuchet-Lourenco et al., 2011; Everett et al., 2013; Hannoun et al., 2016). Among PML isoforms (PML.I–VI), PML.VI fails to associate with the viral-induced foci due to a lack of exon 7a that contains SUMO-interacting motif (SIM). Moreover, mutations of SIMs in PML.I and PML.IV negatively influenced their recruitment to viral-induced foci. Consistent with the correlation between PML recruitment to viral genomes and viral repression, the relief in PML-depleted cells of the restriction of ΔICP0 HSV-1 was reversed following reconstitution of WT PML.I, but not PML.I SIM mutants. Similar to PML SIM mutants, PML.I and PML.IV carrying a single or multiple mutation(s) at major SUMOylation sites (K65, K160, K490, and K616) were less efficiently recruited to viral genome foci. Sp100 and Daxx recruitment to viral foci were also found to occur in a SIM-dependent manner (Cuchet-Lourenco et al., 2011, 2012). Correspondingly, depletion of Ubc9, the sole SUMO E2 conjugating enzyme, impaired the recruitment of PML-NB restriction factors to ΔICP0 HSV-1 genomes and enhanced plaque formation (Boutell et al., 2011). These studies collectively demonstrated the key role of the host SUMO pathway in the regulation of PML-NB-mediated intrinsic antiviral immunity.

#### Protein Inhibitor of Activated STAT (PIAS) 1 and 4

PIAS is a family of SUMO E3 ligases that facilitate the third enzymatic step of the SUMO pathway (Rytinki et al., 2009). PIASs have been mainly known for their role as suppressors of innate immune signaling. Recently, novel roles for them as intrinsic restriction factors that combat HSV-1 infection have been identified (Brown et al., 2016; Conn et al., 2016). Among the members of the PIAS family, PIAS1 is the only type that has been shown to be a permanent constituent PML-NB protein (Brown et al., 2016). However, both PIAS1 and PIAS4 play a key role in mediating the intrinsic antiviral response to HSV-1 infection (Brown et al., 2016; Conn et al., 2016). By conducting the classic plaque edge assay, it was found that both PIAS1 and PIAS4 were recruited to the infecting viral genomes at the nuclear periphery of newly infected cells in a SIM-dependent manner. Depletion of PIAS1 or PIAS4 individually enhanced the plaque formation of ΔICP0 HSV-1 while WT HSV-1 plaque formation remained unaffected. Simultaneous depletion of PIAS1 and PIAS4, or PML with either one of the PIASs, additively enhanced the plaque formation of ΔICP0 HSV-1, demonstrating that PIAS1, PIAS4, and PML act cooperatively to mediate the intrinsic antiviral response to HSV-1 infection (Brown et al., 2016; Conn et al., 2016).

#### DNA Damage Response Proteins

The main function of the DDR machinery is to maintain the integrity of the host genomic DNA and ensure the fidelity of replication (Jackson and Bartek, 2009). Ring finger protein-8 and -168 (RNF8 and RNF168, respectively) play key roles in recruiting repair factors to sites of DNA damage (Mailand et al., 2007; Doil et al., 2009). During WT HSV-1 infection, RNF8 and RNF168 are targeted for degradation by the viral E3 ubiquitin ligase ICP0 (Lilley et al., 2010). In the absence of ICP0, however, RNF8 and RNF168 in addition to other DNA repair factors (e.g., p53-binding protein 1, and breast cancer-1) have been shown to be redistributed to sites adjacent to newly infecting viral genomes in cells at the edge of developing plaques (Lilley et al., 2011). This recruitment phenotype occurs independently of the presence of PML and Daxx, and it raises the hypothesis that RNF8 and RNF168 are involved in the intrinsic antiviral response. Indeed, regarding ΔICP0 HSV-1 infection of RNF8−/− mouse embryonic fibroblasts, the plaque-forming efficiency and viral gene expression were clearly reduced following transduction with retrovirus expressing human RNF8 compared to transduction with an empty retrovirus vector. Initial reduction in the gene expression of WT virus was observed as a result of RNF8 ectopic expression, but this effect was recovered as the infection progressed, probably due to ICP0-induced degradation of RNF8 (Lilley et al., 2010, 2011). These studies added RNF8 and RNF168 to the growing list of host restriction factors that mediate the intrinsic antiviral response to HSV-1 infection.

#### Epigenetic Regulators

Several studies have reported associations of the viral genomes with nucleosomes and their components during viral latency (Deshmane and Fraser, 1989; Maroui et al., 2016; Cohen et al., 2018). The nucleosome is the basic unit of chromatin and is composed of 146 bp of DNA wrapped around a histone octamer (an H3-H4 histone protein tetramer that interacts with two H2A-H2B dimers via two H2B–H4 associations). Linker histone (H1) variants also bind to nucleosomes and mediate chromatin compaction. These epigenetic regulators were found to confer an intrinsic antiviral response to HSV-1 lytic infection (Knipe, 2015).

As early as 1 h post-infection (hpi), core histones (H3) with repressive marks [e.g., H3 lysine 9-trimethylation (H3K9me3) and H3 lysine 27-trimethylation (H3K27me3)] were associated with the incoming viral genomes in a manner that increased over time (Kent et al., 2004; Oh and Fraser, 2008; Lee et al., 2016). Independent studies demonstrated mobilization and association of core histones (H2B and H4) and linker histones (H1 variants) with the vDNA upon entry into the nucleus. Histone mobilization occurs independently of viral gene expression. Nevertheless, the expression of IE and E genes, but not vDNA replication or L gene expression, promotes this process (Conn et al., 2008, 2011, 2013). In the absence of viral countermeasures (e.g., ICP0 and VP16), recruitment of repressive histones induced viral genome chromatinization and silencing. The histone chaperone HIRA and chromatin remodeling protein ATRX were found to be important in this process (Rai et al., 2017; Cabral et al., 2018). HIRA is localized to PML-NBs upon viral infection and deposits H3 variants (H3.3) onto incoming vDNA, and ATRX stably maintains vDNA heterochromatin, leading to intrinsic restriction of viral replication (Rai et al., 2017; Cabral et al., 2018).

Epigenetic regulators are also involved in impairing the viral infection during the transition from IE to E protein expression (Gu et al., 2005; Gu and Roizman, 2007). Indeed, many infected cells express IE proteins during ΔICP0 HSV-1 infection but the infection becomes stalled at this stage (Everett et al., 2004a). This phenotype is partly mediated by the RE1-silencing transcription factor (REST)/CoREST/histone deacetylases (HDAC) nuclear repressor complex. Inhibitors of HDAC and a mutant CoREST lacking the HDAC1 binding site both enhanced the transition of viral gene expression and viral replication in the absence of ICP0, again highlighting the key role of epigenetic regulators as intrinsic restriction factors that can combat HSV-1 infection (Gu et al., 2005; Gu and Roizman, 2007).

### Innate Immunity

Innate immunity, unlike constitutive intrinsic immunity, is mediated by cellular proteins induced in response to IFNs, a family of proinflammatory cytokines that play central antiviral roles during HSV-1 infection (Mossman and Ashkar, 2005; Chew et al., 2009; Knipe, 2015). IFNs are classified into three main types depending on the receptors utilized for signaling: (i) IFN type I (IFN-I) comprises IFNα, β, ε, κ, and ω, which utilize IFNα receptors 1 and 2 (IFNAR1/2) (Gonzalez-Navajas et al., 2012), (ii) IFN type II (IFN-II) comprises IFNγ, which utilizes the IFNγ receptor (IFNGR) (Platanias, 2005), and (iii) IFN type III (IFN-III) comprises IFNλ1, λ2, and λ3 (IL-28A, IL28B, and IL29, respectively), which utilize the IFNλ receptor (IFNLR), also known as IL 28 receptor α (IL-28Rα) and IL-10 receptor β (IL-10Rβ) (Zanoni et al., 2017). Some cell types can produce and respond to more than one type of IFN while others are predominantly responsible for a specific type of IFN expression and signaling (Lee and Ashkar, 2018; Lazear et al., 2019). The induction of an IFN response during viral infections, including HSV-1 infection, involves two phases: (a) the first phase is initiated following sensing of pathogen-associated molecular patterns (PAMPs; e.g., viral particles or viral replication products) by pattern recognition receptors (PRRs), leading to the production of IFNs, and (b) the second phase starts when the secreted IFNs bind to their cognate receptors and subsequently activate IFN signaling cascades, resulting in the induction of IFN-stimulated genes (ISGs) whose products establish an antiviral state in the infected cells and neighboring uninfected cells to control the infection (Knipe, 2015; Kurt-Jones et al., 2017).

#### Importance of IFN Response in Controlling HSV-1 Replication

The role of IFN-I in controlling HSV-1 infection has been extensively studied. Historically, the resistance and susceptibility of different mouse strains to HSV-1 infection were linked to their abilities and efficiencies to induce an IFN-I response (IFNα and IFNβ) (Lopez, 1975; Zawatzky et al., 1982; Halford et al., 2004). Increased viral replication, severe pathogenesis, and reduced survival rates have been observed in mice lacking IFNAR in comparison to WT controls (Leib et al., 1999; Luker et al., 2003). Several *in vitro* studies also highlighted the important role of IFN-I in controlling the replication, spread, and cytopathic effect of HSV-1 (Domke-Opitz et al., 1986; Sainz and Halford, 2002; Rosato and Leib, 2014). Further studies discovered several PRRs and cellular factors required for IFN-I production, characterized the related signaling cascades, and identified ISG products with antiviral properties as well as the viral evasion strategies (reviewed below).

The IFN-II (IFNγ) signaling pathway plays crucial roles in controlling and minimizing the pathogenesis of HSV-1 infection during lytic infection (Bigley, 2014). Mice lacking IFNGR were more vulnerable to HSV-1 infection and had a higher mortality rate than WT mice (Cantin et al., 1995, 1999; Minami et al., 2002). IFNγ was found to synergize with IFN-I during HSV-1 infection, leading to a dramatic reduction in viral replication (Sainz and Halford, 2002; Vollstedt et al., 2004). Correspondingly, mice lacking both IFNAR and IFNGR had increased susceptibility to HSV-1 infection in comparison to mice lacking either one of the receptors individually (Luker et al., 2003). It is also known that IFNγ links the host innate and adaptive immune responses. It stimulates the expression of major histocompatibility complex class I to enhance antigen presentation to CD8^+^ T cells, which plays a key role in the maintenance of viral latency. Indeed, mice lacking IFNGR displayed higher levels of viral gene expression and replication during reactivation than WT mice (Shaw et al., 1985; Cantin et al., 1999).

IFN-III (IFNλ1–3), the most recently discovered member of the IFN family, has unique receptors (IFNLRs) but utilizes the same signaling cascade as IFN-I (Zanoni et al., 2017; Lazear et al., 2019). Few studies have addressed the role of IFNλ during HSV-1 infection. Exogenous treatment of primary human astrocytes and neurons with IFNλ inhibited the viral gene expression and viral protein synthesis, probably by stimulating the induction of endogenous IFN-I production and ISG expression (Li et al., 2011). Similarly, the subset of plasmacytoid dendritic cells (pDCs) that can produce IFNλ in response to HSV-1 infection were shown to be associated with a higher level of IFNα and a more efficient antiviral response in comparison to cells that failed to produce IFNλ (Yin et al., 2012). Moreover, pretreatment of pDCs with IFNλ resulted in enhanced IFNα production following HSV-1 infection (Yin et al., 2012). These findings indicate that IFN-λ is an autocrine signaling factor that rapidly primes an IFN-I antiviral response in HSV-1-infected cells (Li et al., 2011; Yin et al., 2012). However, the underlying mechanism(s) of IFN-λ-mediated antiviral immunity remains far from being fully understood and requires further study.

#### Sensing and Recognition of HSV-1 by PRRs

**FIGURE 4:**
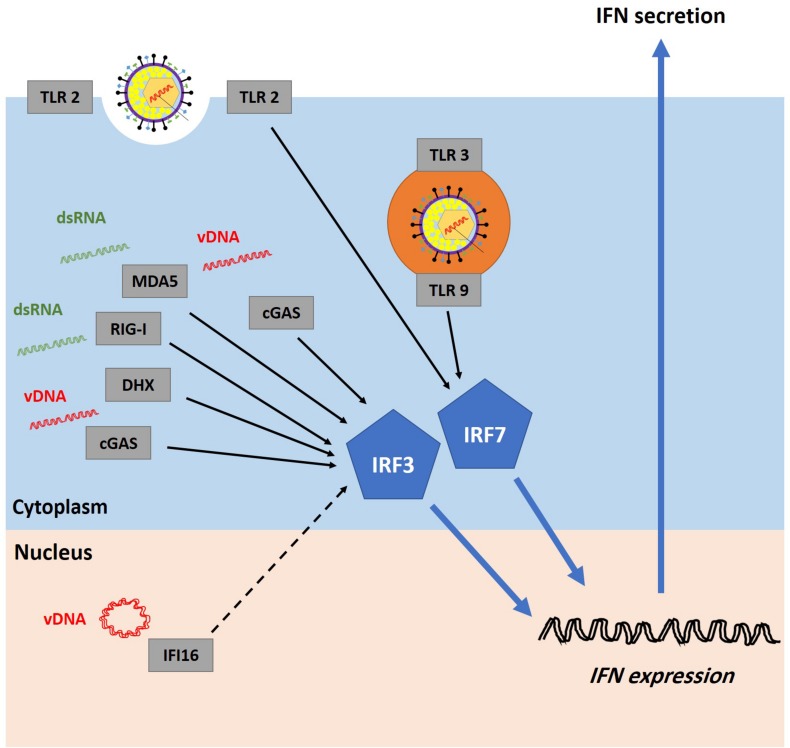
Recognition of HSV-1 infection by pattern recognition receptors (PRRs). Host cells are equipped with several PRRs that can recognize virion components (e.g., glycoprotein and vDNA) and structures accumulated during vDNA replication (e.g., dsRNA). Examples of PRRs include cyclic guanosine monophosphate-adenosine monophosphate synthase (cGAS), DExD/H-box helicases (DHX), melanoma differentiation-associated protein 5 (MDA5), retinoic acid-inducible gene I (RIG-I), and toll-like receptors (TLRs). It remains highly controversial whether interferon-gamma-inducible protein 16 (IFI16) can sense incoming vDNA in the nucleus (the dashed line represents uncertainty). PRRs signal through the stimulator of interferon genes (STING)-TANK-binding kinase 1 (TBK1)-IFN regulatory factor 3 and 7 (IRF3/7) pathway to induce interferon (IFN) production. Adapted with permission from Alandijany (2018).

The activation of the first phase of the IFN response is dependent on the ability of PRRs to recognize PAMPs in the infected cells. The interactions between PRRs and their viral ligands leads to the activation of TANK-binding kinase 1 (TBK1) in fibroblasts or inhibitor of nuclear factor kappa B (NFκB) epsilon (IKKε) in immune cells. These protein kinases induce the phosphorylation and activation of IFN regulatory factor 3 and 7 (IRF3 and IRF7) which, in cooperation with other transcription factors, bind to IFN gene promoters and stimulate IFN secretion (Chew et al., 2009). Numerous PRRs have been identified (Figure 4). They can recognize and sense virion components (e.g., viral glycoprotein and vDNA) as well as viral replication intermediates and products (e.g., cytosolic dsRNA) (Paludan et al., 2011). However, viral nucleic acid is likely the most potent PAMP inducer of host innate immunity (Paludan and Bowie, 2013; Knipe, 2015; Komatsu et al., 2016).

##### Toll-like receptors

Toll-like receptors (TLRs) were among the first PRRs to be studied and characterized. They can be found at the plasma membrane (e.g., TLR1, TLR2, and TLR4) and within endosomes (e.g., TLR3, TLR7, TLR8, and TLR9). TLR2, TLR3, and TLR9 have been shown to be critical in controlling HSV-1 replication and dissemination, and their functions appear to be redundant and cell type-specific (Krug et al., 2004; Rasmussen et al., 2007; Zhang et al., 2007; Sorensen et al., 2008; Guo et al., 2011; Cai et al., 2013; Mork et al., 2015; Zhang and Casanova, 2015; Uyangaa et al., 2018). It has been proposed that TLRs on the plasma membrane detect viral glycoproteins while TLRs within endosomes sense viral nucleic acids, though this has not been formally investigated during HSV-1 infection (Ma and He, 2014).

An initial *in vitro* study demonstrated TLR9-dependent IFN-I production in HSV-1-infected pDCs (Krug et al., 2004). A few years later, the antiviral role of TLR9 during HSV-1 infection both *in vivo* and *ex vivo* was confirmed (Rasmussen et al., 2007). WT and TLR9−/− mice were infected with HSV-1, and the level of IFN-I production was measured in the serum and in isolated conventional DCs, pDCs, macrophages, and fibroblasts. In WT mice, IFN-I was detectable in the serum at 8 hpi, peaked at 16 hpi, and diminished at 48 hpi. The initial induction of IFN-I expression (8 hpi) was dependent on the presence of TLR9. However, no difference was noticed at 16 hpi, suggesting a redundant role for TLR9 in cytokine production during HSV-1 infection. Consistently, viral infection induced IFN-I production in all isolated cell types, and only pDCs required TLR9 for this process (Rasmussen et al., 2007). TLR9 was found to synergize with the plasma membrane TLR2 to control viral replication and dissemination to the central nervous system (CNS), although other studies have suggested that TLR2 activation can be immunopathological (Kurt-Jones et al., 2004; Sarangi et al., 2007; Sorensen et al., 2008; Uyangaa et al., 2018). In addition to TLR2 and TLR9, the presence of functional TLR3 is believed to be key for an efficient antiviral response to HSV-1 infection. Several studies demonstrated that herpes encephalitis is associated with TLR3 deficiency or lack of a functional TLR3 (Zhang et al., 2007; Guo et al., 2011; Zhang and Casanova, 2015; Mielcarska et al., 2018). Collectively, these data highlight the important antiviral role of TLRs during HSV-1 infection.

##### Retinoic acid-inducible gene I (RIG-I) like receptors (RLRs) and DExD/H-box helicases (DHXs)

Melanoma differentiation-associated protein 5 (MDA5) belongs to the RLR family. It serves as a cytosolic double-stranded RNA (dsRNA) sensor and mediates cytokine signaling through its adaptor protein, mitochondrial antiviral signaling protein (MAVS) (Yoneyama et al., 2005). MDA5 preferentially recognizes long dsRNA (>1000 bp) and large RNA aggregates (Kato et al., 2008; Li et al., 2009; Pichlmair et al., 2009). Many viruses including HSV-1 produce dsRNA during replication (Weber et al., 2006). Research demonstrated that HSV-1 infection induced cytokine and chemokine production such as IFN-I, IFN-III, tumor necrosis factor α (TNFα), and C-C motif chemokine ligand 5) in human monocyte-derived macrophages (Melchjorsen et al., 2006, 2010). This process required virus entry and replication but occurred independently of TLR2 and TLR9. MAVS or MDA5 knockdown led to significantly lower levels of HSV-1-induced IFN-I and IFN-III expression, while RIG-I knockdown did not affect this process (Melchjorsen et al., 2006, 2010). However, an independent study showed that RIG-I and MDA5 synergistically contribute to innate immune recognition of HSV-1 infection and upregulation of IFN-I genes (IFNα and IFNβ) in mouse embryonic fibroblasts and HeLa cells (Choi et al., 2009). Interestingly, this transfection-based study demonstrated that RIG-I and MDA5, known to be RNA sensors, serve as cytosolic DNA sensors and mediate IFN-I expression by activating the IRF3 pathway. Of note, RIG-I and MDA5 also belong to the DHX family. Further research identified other DHXs, namely DHX9 and DHX36, as cytosolic DNA sensors (Kim et al., 2010). The presence of DHX9 and DHX36 was crucial for efficient induction of cytokine and chemokine expression in HSV-1-infected pDCs. While DHX9-mediated sensing induced NFκB activity and TNFα expression, DHX36 activation was associated with IRF7 nuclear translocation and IFNα production (Kim et al., 2010).

##### Cyclic guanosine monophosphate-adenosine monophosphate (cyclic GMP-AMP, or cGAMP) synthase

Cyclic guanosine monophosphate-adenosine monophosphate synthase (cGAS), a member of the nucleotidyltransferase family, has been identified as a cytosolic DNA sensor during HSV-1 infection (Sun et al., 2013). Following the recognition of cytosolic DNA, cGAS promotes cGAMP production, which interacts with stimulator of interferon genes (STING), leading to IRF3 activation and IFNβ production (Wu et al., 2013). The post-translational modification status of cGAS plays a key role in its DNA sensing ability and innate immunity induction (Cui et al., 2017; Wang et al., 2017). cGAS is SUMOylated at different sites: K335, K372, and K382, which suppresses its DNA-binding capacity. Sentrin-specific protease 7 (SENP7) deSUMOylates cGAS and primes it for activation, leading to efficient IRF3-dependent induction of innate immunity. Knockdown of SENP7 in infected mice impaired IFN secretion and ISG expression, rendering them more vulnerable to HSV-1 infection (Cui et al., 2017). It was also demonstrated that RNF185, the first E3 ubiquitin ligase identified for cGAS, binds and promotes polyubiquitination of cGAS at K27. Similar to SENP7 knockdown, RNF185 knockdown negatively influenced cGAS activity and innate immunity induction during HSV-1 infection (Wang et al., 2017).

##### Interferon-gamma-inducible protein 16

Interferon-gamma-inducible protein 16 (IFI16), which belongs to the pyrin domain and two DNA-binding hematopoietic interferon-inducible nuclear proteins with 200-amino acids repeat domains (PYHIN) protein family, was initially reported as a cytosolic DNA sensor of transfected foreign DNA derived from the HSV-1 genome (Unterholzner et al., 2010). Short-hairpin RNA-mediated depletion of IFI16 or the mouse ortholog of IFI16 (p204) inhibited IFNβ production in response to DNA transfection. Notably, stimulation of IFI16-mediated sensing was dependent on the foreign DNA length and structure but occurred independently of its nucleotide content (Unterholzner et al., 2010). An independent study also highlighted the key role of IFI16 as a cytosolic DNA sensor in HSV-1-infected macrophages (Horan et al., 2013). HSV-1 capsid proteins are ubiquitinated and targeted for proteasomal degradation in the cytoplasm of infected cells, exposing vDNA to IFI16-mediated sensing and innate immunity induction. At permissive and non-permissive temperatures, TsB7 (an HSV-1 temperature-sensitive mutant whose capsids accumulate in the cytoplasm and fail to release the viral genomes due to a defect in VP1-2 function) induced IFNβ and ISG56 to equivalent levels observed during WT HSV-1 infection. Localization of IFI16 near the TsB7 mutant DNA was also unaffected at the non-permissive temperature. Therefore, it was concluded that the nuclear import of viral genomes in human macrophages was not required for the induction of IFI16-mediated innate immunity (Horan et al., 2013).

Given that IFI16 is predominantly localized to the nucleus of many types of cells (e.g., fibroblast, endothelial, and epithelial cells), subsequent studies investigated whether IFI16 can serve as a nuclear DNA sensor. A multiphasic and dynamic subnuclear redistribution of IFI16 has been identified in HSV-1-infected fibroblasts (Orzalli et al., 2012; Cuchet-Lourenco et al., 2013; Diner et al., 2015; Everett, 2015). As early as 0.5–1 hpi, IFI16 puncta were transiently formed on the nuclear periphery of newly infected cells at the edge of developing plaques. As the infection progressed (approximately 3–4 hpi), IFI16 puncta were observed to assemble in the nucleoplasm of the infected cells. Soon after, these IFI16 signals were lost in WT HSV-1-infected cells, but they remained stable during ΔICP0 HSV-1 infection. This phenotype is believed to be crucial for IFI16-dependent IFNβ production and limitation of viral replication, although it also has a role in IFN-independent intrinsic repression of viral genomes (Orzalli et al., 2012, 2013; Johnson et al., 2014; Diner et al., 2015). Importantly, blocking of vDNA release into the nucleus using tosyl phenylalanyl chloromethyl ketone substantially inhibited the induction of IFNβ and ISG54 following infection, demonstrating that vDNA accumulation in the nucleus of fibroblasts, unlike in macrophages, is required for the induction of the innate immune response (Orzalli et al., 2012). Through its positively charged hematopoietic interferon-inducible nuclear proteins with 200-amino acids repeats (HIN) domain, nuclear IFI16 interacts with the sugar-phosphate backbone of foreign dsDNA, which releases the pyrin domain from its autoinhibited state (Jin et al., 2012). Following acetylation of its nuclear localization signal by acetyltransferase p300, activated IFI16 translocates to the cytoplasm and activates the STING pathway. STING associates with TBK1 and promotes IRF3 phosphorylation and nuclear translocation, eventually leading to IFNβ gene upregulation and cytokine production (Jin et al., 2012; Ansari et al., 2015).

#### Induction of ISG Products

**FIGURE 5:**
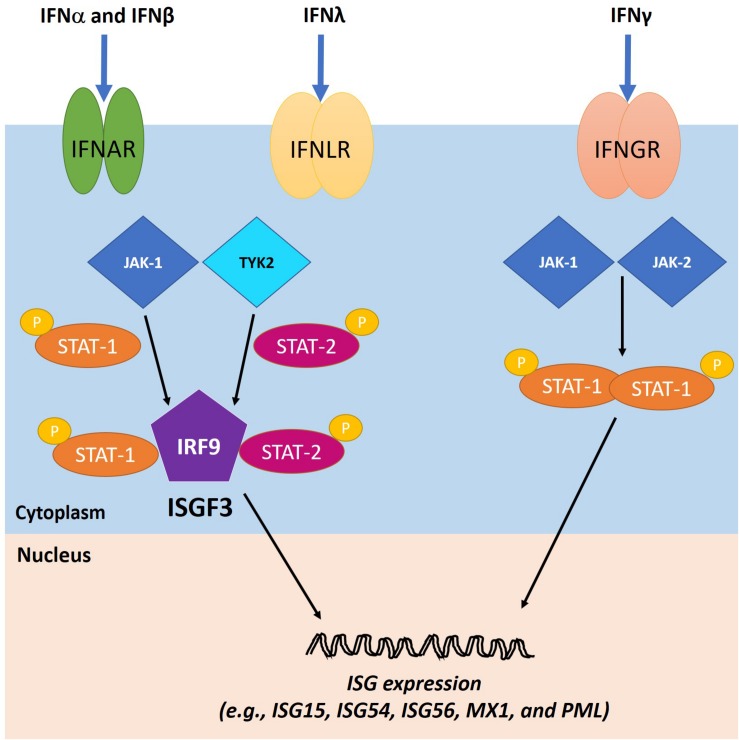
IFN signaling cascades. The IFN type I (IFN-I) and III (IFN-III) bind to their cognate receptors, IFNα receptors (IFNAR) and IFNλ receptors (IFNLR), respectively, on the cell surface, leading to the activation of Janus kinase 1 (JAK-1) and tyrosine kinase 2 (TYK-2). As a result, activated signal transducers and activators of transcription 1 and 2 (STAT1 and STAT2, respectively) are phosphorylated and bind to IFN regulatory factor 9 (IRF9) to form ISGF3, which translocates to the nucleus to induce the expression of ISGs. The IFN type II (IFN-II) signaling cascade is triggered when IFNγ binds to IFNγ receptor (IFNGR) and activates JAK1 and Janus kinase 2 (JAK2). Consequently, STAT1 molecules are phosphorylated and localized to the nucleus to transactivate ISG expression. Adapted with permission from Alandijany (2018).

The second phase of the IFN response starts when secreted IFNs bind to their cognate receptors. IFN-I and IFN-III, although utilizing distinct receptors, signal through the same pathways (Lazear et al., 2019). They activate Janus kinase 1 (JAK-1) and tyrosine kinase 2 (TYK-2), which subsequently induce the phosphorylation and accumulation of activated signal transducers and activators of transcription 1 and 2 (STAT-1 and STAT-2, respectively). Interaction between STAT1, STAT2, and IRF9 leads to the formation of the IFN-stimulated gene factor 3 (ISGF3) complex at ISG promoters, which induces their expression (Lazear et al., 2019). The IFN-II signaling cascade is triggered when IFNγ binds to IFNGR, followed by assembly of the IFNγ-IFNGR-JAK1-JAK2 complex. Activation of JAK1 and JAK2 induces IFNGR phosphorylation and STAT1 docking site formation. STAT1 molecules are first recruited to the complex and phosphorylated, and they then become dissociated and are eventually translocated to the nucleus where they act as ISG transactivators (Lee and Ashkar, 2018). The IFN signaling pathways function in both autocrine and paracrine fashions in order to inhibit viral replication in the infected cells and protect neighboring cells from infection (Figure 5).

Several cellular ISG products with antiviral effects against HSV-1 infection have been identified. Most of the studies have relied on utilizing viral mutants that lack IFN antagonists. For instance, the ISG products viperin, tetherin, and zinc finger antiviral protein (ZAP) have been found to restrict HSV-1 infection. However, their antiviral activities rely on the absence of HSV-1 virion host shutoff (vhs) protein (Zenner et al., 2013; Shen et al., 2014; Su et al., 2015). Indeed, ectopic expression of viperin, tetherin, and ZAP reduced the viral yield of a vhs-null mutant but not the WT virus, while depletion of these proteins enhanced the viral yield of the vhs-null mutant. The vhs protein was found to target the mRNAs of these ISG products for degradation, and it consequently counteracted their antiviral properties. Protein kinase R and 2′-5′-oligoadenylate synthetase were also shown to confer antiviral immunity to HSV-1 infection in a process that is efficiently counteracted by the viral protein Us11 (Sanchez and Mohr, 2007; Lussignol et al., 2013). Only two ISG products have been identified as antiviral factors during WT HSV-1 infection, namely ISG15 and MxB (Lenschow et al., 2007; Crameri et al., 2018). An *in vivo* study suggested that the presence of ISG15, a ubiquitin-like molecule that is rapidly induced following viral infection, is key for mediating the IFN antiviral response against HSV-1 infection. In comparison with WT mice, ISG15-deficient mice showed increased susceptibility to HSV-1 infection and a decreased survival rate. However, the mechanism underlying this restriction process remains unknown (Lenschow et al., 2007). Recently, MxB was shown to restrict the HSV-1 life cycle in IFN-pretreated cells by interfering with vDNA delivery to the nucleus (Crameri et al., 2018). These studies shed light on ISG products with antiviral effects against HSV-1 infection, but this crucial area of research remains largely understudied, especially regarding WT HSV-1 infection, which is known to be impaired by IFN pretreatment of cell lines and animal models (Domke-Opitz et al., 1986; Taylor et al., 1998; Mossman et al., 2000; Sainz and Halford, 2002; Everett and Orr, 2009).

#### Temporal Regulation of Host Intrinsic and Innate Intracellular Immunity

It is clear that both intrinsic (constitutive) and innate (inducible) antiviral responses play key roles in the intracellular restriction of HSV-1 infection. Rapid recognition of viral nucleic acids is key for both arms of immunity (Paludan and Bowie, 2013; Knipe, 2015; Komatsu et al., 2016). Until recently, several questions had remained unanswered with regard to how intrinsic and innate immune responses are regulated. For example, (1) are they simultaneously or sequentially triggered in response to infection, (2) do they similarly or distinctly impair viral replication, and (3) does the permissiveness and vulnerability of certain cell types to ΔICP0 HSV-1 infection correlate with the lack of ability to mount an efficient intrinsic and/or innate immune response in these cells?

One of the main reasons why the temporal regulation of intrinsic and innate immunity remains poorly defined is the fact that most microscopy-based studies of host factor recruitment to HSV-1 genomes have utilized indirect methods to detect vDNA (e.g., immunostaining or fluorescent tagging of vDNA-binding proteins) (Everett et al., 2003, 2004b; Everett and Murray, 2005; Orzalli et al., 2015; Diner et al., 2016; Komatsu et al., 2016). These methods allowed identification of many intrinsic and innate immune factors. However, as these approaches necessitate the onset of viral gene expression, our understanding of the viral–host interactions that occur immediately upon nuclear entry of viral genomes (prior to the expression of viral proteins) was limited. The onset of viral gene expression may also displace host factors recruited or bound to viral genomes. Several studies have utilized direct methods for vDNA detection (e.g., FISH and bromodeoxyuridine-labeling of vDNA) (Maul et al., 1996; Everett et al., 2007; Glauser et al., 2007). However, these methods require harsh denaturation conditions and substantial sample processing, which can be incompatible with immunofluorescent staining of host factors (Jensen, 2014). Moreover, these experiments were conducted under high MOI conditions (≥10 PFU/cell) due to the technical difficulties associated with detection of low genome copy numbers. Utilizing high MOI conditions is suboptimal or even unsuitable for studying the regulation of intrinsic and innate immunity, given that intrinsic immunity has a threshold MOI above which it becomes saturated and no longer effective (Everett et al., 2004a). In addition, high MOI conditions trigger an IFN response, and several intrinsic immune factors (e.g., PML and Sp100) are considered as ISG products, which makes it difficult to distinguish their intrinsic from their innate antiviral roles (Lavau et al., 1995; Grotzinger et al., 1996).

Recently, to track the subcellular localization of vDNA, there has been an increase in the use of pyrimidine deoxynucleotide analog labeling of HSV-1 DNA in combination with click chemistry (Wang et al., 2013; Sekine et al., 2017; Alandijany et al., 2018; Cabral et al., 2018; Sun et al., 2019). This technique, which is not detrimental to viral infectivity, enables direct visualization of input viral genomes under low MOI conditions (as low as 0.1 PFU/cell) immediately upon nuclear entry (∼30 min post-virus addition). Furthermore, it is sensitive and specific to vDNA and compatible with indirect immunofluorescence staining protocols, providing a valuable method to investigate the temporal recruitment of intracellular immune regulators to infecting viral genomes at single-cell and single-molecule levels.

Utilizing this technique, it was found that PML-NB restriction factors (e.g., PML, SP100, Daxx, and ATRX) were rapidly recruited to infecting viral genome foci upon the entry of the vDNA into the nucleus of infected human foreskin fibroblasts (∼30 min post-virus addition) (Alandijany et al., 2018; Cabral et al., 2018). This process occurred in a PML-dependent manner and led to genome entrapment and silencing within PML-NBs. Interestingly, genome entrapment was observed during both WT and ΔICP0 HSV-1 infection. However, during WT infection, ICP0 induced PML degradation and the dispersal of the PML-NB restriction factors, highlighting the importance of ICP0 in the release of viral genomes entrapped within PML-NBs to stimulate the onset of lytic HSV-1 replication. In contrast, during ΔICP0 HSV-1 infection, vDNA remained stably entrapped within PML-NBs, leading to repression of viral gene expression and restriction of plaque formation. Importantly, the host PRR and DNA sensor IFI16 was not stably recruited to vDNA entrapped within PML-NBs, and ISG expression was not induced under low MOI conditions that did not saturate the PML-NB intrinsic host defenses (Alandijany et al., 2018). An independent study on MRC-5 human embryonic lung fibroblasts that utilized mass spectrometry analysis supported these findings regarding the WT virus, demonstrating the association of PML, but not IFI16, with WT viral genomes prior to viral gene expression immediately upon nuclear entry (Dembowski and DeLuca, 2018). Instead, IFI16 was associated with the viral genomes that successfully initiated gene expression, as demonstrated by ICP4 expression. Data from these studies indicate that vDNA entry into the nucleus alone stimulates the recruitment of intrinsic restriction factors (such as PML) to the infecting genomes, but it is not sufficient for nuclear PRRs to recognize vDNA (Alandijany et al., 2018; Dembowski and DeLuca, 2018).

**FIGURE 6:**
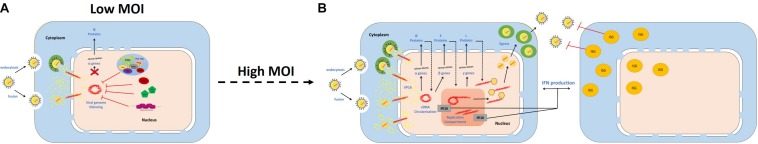
Temporal regulation of intrinsic and innate immunity during HSV-1 infection. Schematic diagrams demonstrate the sequential recruitment of intrinsic restriction factors and pattern recognition receptors (PRRs) to viral DNA (vDNA), and the associated antiviral effects. **(A)** Under low multiplicity of infection (MOI) conditions, intrinsic restriction factors are recruited to vDNA upon nuclear entry to induce viral genome silencing. **(B)** Under high MOI conditions, escape from the intrinsic immune response and initiation of vDNA replication enables PRR recruitment and induction of interferon (IFN) production. Innate immunity constricts viral propagation and limits the spread of infection.

Saturation of intrinsic host defenses under higher MOI conditions (1 PFU/cell) stimulated the stable recruitment of IFI16 to infecting viral genomes, and induced ISG expression in an IFI16- and JAK-dependent manner. The induction of this innate immune response was dependent on the onset of viral gene expression and vDNA replication, as treatment of infected cell monolayers with phosphonoacetic acid (a vDNA polymerase inhibitor) inhibited ISG induction in a dose-dependent manner (Alandijany et al., 2018). Unlike intrinsic immune factor depletion, inhibition of JAK signaling failed to relieve the plaque formation defect of ΔICP0 HSV-1, and instead significantly enhanced the virus yield. These findings led to the conclusion that the intrinsic and innate arms of intracellular immunity are temporally and functionally distinct from one another (Alandijany et al., 2018). Intrinsic immunity acts immediately upon vDNA delivery to the nucleus to induce viral genome silencing. Escape from this immune response and initiation of vDNA replication trigger innate immunity in the infected and neighboring uninfected cells, which constricts viral propagation and limits the spread of infection (Figure 6). Cell types such as U2OS and SAOS, which fail to efficiently recruit intrinsic restriction factors to viral genomes and/or to induce a robust innate immune response, are highly permissive to HSV-1 infection even in the absence of ICP0 (Deschamps and Kalamvoki, 2017; Alandijany et al., 2018).

Nevertheless, the authors of previous studies that utilized ultraviolet (UV)-inactivated WT virus, WT virus in the presence of cycloheximide, or replication-incompetent viral mutants defective in multiple genes argued that initiation of vDNA replication is not required for innate immunity induction (Nicholl et al., 2000; Preston et al., 2001; Eidson et al., 2002; Collins et al., 2004). On the surface, these findings appear contradictory to the recent report described above (Alandijany et al., 2018). However, it is important to note that higher MOI conditions (5–50 PFU/cells) were used in these studies. In some cases, information about the particle-to-PFU ratio of viral stocks was missing, which is particularly critical in the case of viral mutants as they are usually associated with incredibly high particle-to-PFU ratios (Everett, 1989; Preston et al., 2001; Collins et al., 2004; Everett et al., 2004a). Additionally, UV inactivation of HSV-1 may have detrimental effects on viral capsids (e.g., degradation or permeabilization), as observed for other viruses (Miller and Plagemann, 1974; De Sena and Jarvis, 1981; Smirnov et al., 1983). The experimental settings used might be problematic and physiologically irrelevant, as they may deliver or generate PAMPs (e.g., accumulation of a large number of capsids, premature DNA release in the cytoplasm, and aggregation of high-order vDNA structures in the nucleus) that allow PRR detection.

More recent studies proposed that vDNA entry into the nucleus is not required for innate immunity induction in immune cells (e.g., macrophages) (Horan et al., 2013; Sun et al., 2019). Both IFI16 and cGAS can sense vDNA in the cytoplasm in these cells, leading to induction of an IFN response and the elimination of the infecting viral genomes. This is quite intriguing as viral genomes should, in theory, remain encapsulated within capsids during cytoplasmic transportation and only be released upon ejection through the nuclear pores, which makes vDNA inaccessible to DNA sensors. One explanation is that HSV-1 capsids are targeted for proteasomal degradation in the cytoplasm of infected macrophages, allowing IFI16 sensing of naked vDNA (Horan et al., 2013). Independent of proteasomal degradation, premature release of vDNA into the cytoplasm of infected monocytes has been reported (Sun et al., 2019). This premature release is believed to enable cGAS-mediated sensing of viral genomes, induction of an IFN response, and clearance of the cytosolic viral genomes and capsids (Sun et al., 2019). Importantly, however, high MOI conditions (10 PFU/cell, and sometimes up to 100 PFU/cell) were utilized in these studies (Horan et al., 2013; Sun et al., 2019). Thus, the discrepancies in observations among the studies with regard to cellular and viral requirements underlying pathogen sensing and innate immunity induction may again be due to the MOI conditions used. It is also important to be aware that immune and non-immune cells differ strikingly in their abilities to mount an IFN response to viral infections. Hence, it is possible that the temporal regulation of intrinsic and innate immunity observed in fibroblasts does not apply in macrophages. However, this hypothesis remains to be investigated using a side-by-side comparison involving physiologically relevant low MOI conditions.

## Hsv-1 E3 Ubiquitin Ligase Icp0, a Key Antagonist for Host Intracellular Immunity

**FIGURE 7:**
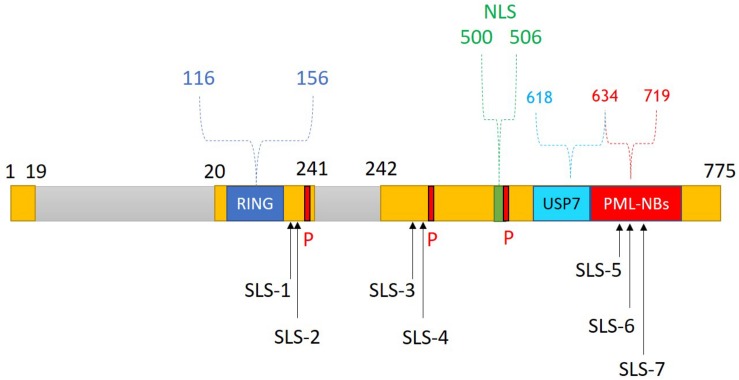
ICP0 structure and functional domains. ICP0 comprises 775 amino acids and is encoded by the *IE-0* gene located within the inverted repeats sequences ab and b′a′. ICP0 is composed of three exons (1–19, 20–241, and 242–775 nucleotides; yellow), and two introns (765 and 136 nucleotides; gray). Several functional domains and interacting motifs have been identified in ICP0. A zinc-binding really interesting new gene (RING) finger domain is located within the N-terminal region of ICP0 (residues 116–156). The C-terminal region contains a nuclear localization signal (NLS; residues 500–506), a ubiquitin-specific protease 7 (USP7)-binding motif (residues 618–634), and sequences required for localization at promyelocytic leukemia protein-nuclear bodies (PML-NBs; residues 634–719). Three major phosphorylation sites (P; 224–234, 365–371, and 508–518) of ICP0 have been identified. ICP0 contains several SUMO interaction motif (SIM)-like sequences (SLS-1 to SLS-7). Reproduced with permission from Alandijany (2018).

**FIGURE 8:**
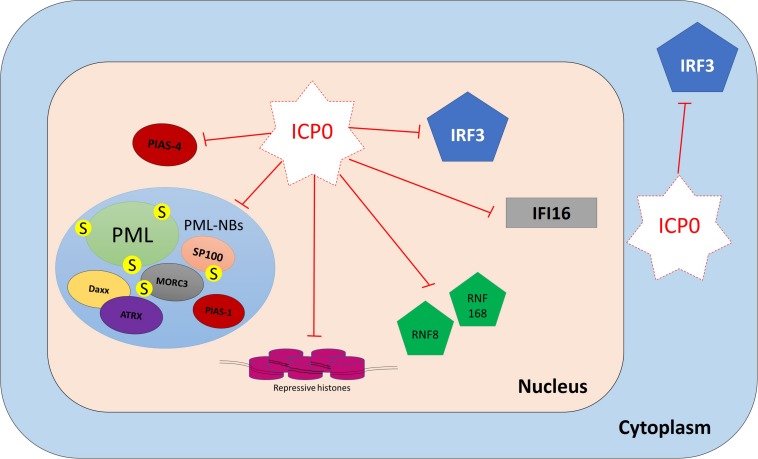
ICP0 counteracts host intrinsic and innate immunity. ICP0 employs multiple strategies to antagonize host intracellular immunity. It targets promyelocytic leukemia protein (PML), speckled 100 kDa (SP100), MORC family CW-type zinc finger 3 (MORC3), protein inhibitor of activated STAT 1 (PIAS1), and interferon-gamma-inducible protein 16 (IFI16) for degradation. It interferes with the recruitment of death domain associated protein (Daxx), alpha thalassemia/mental retardation syndrome X-linked (ATRX), protein inhibitor of activated STAT 4 (PIAS4), and epigenetic repressors to viral DNA (vDNA). It prevents IFN-regulatory factor 3 (IRF3) activity and impairs its function.

HSV-1 has evolved multiple strategies to antagonize and evade the host immune response. In particular, the viral IE protein ICP0 has received significant attention due to its central roles in counteracting both the intrinsic and innate arms of intracellular immunity (Boutell and Everett, 2013; Lanfranca et al., 2014; Gu, 2016; Tognarelli et al., 2019). ICP0 is a multifunctional IE protein that enhances the viral lytic infection and promotes genome reactivation from quiescence/latency (Everett, 2000). It is encoded by the *IE-0* gene (also known as α*0*), which is located within the inverted repeats sequence ab and b′a′ (Wadsworth et al., 1975). Several functional domains and interacting motifs have been identified within ICP0 (Everett, 1988). The zinc-binding really interesting new gene (RING) finger domain located in the N-terminal region between amino acids 116 and 156 within exon 2 is considered the most important functional domain of ICP0 (Figure 7). Indeed, HSV-1 mutants that express a catalytically inactive RING domain had equivalent replication defects as ΔICP0 HSV-1 and failed to reactivate quiescent/latent viral genomes (Everett, 1989; Lium and Silverstein, 1997; Everett et al., 2004a, 2009; Ferenczy et al., 2011; Grant et al., 2012). This RING finger domain confers E3 ubiquitin ligase activity, facilitating the conjugation of ubiquitin molecules to the lysine residues of target proteins and thereby promoting their proteasome-dependent degradation (Boutell et al., 2002; Vanni et al., 2012). Importantly, many ICP0-targeted proteins are key regulators of host intrinsic and innate immunity (Figure 8). Targeting these immune factors using ICP0, directly or indirectly, at the early stages of infection provides a favorable environment for viral replication (Davido et al., 2005; Boutell and Everett, 2013; Gu, 2016).

### ICP0-Mediated Counteraction of Host Intrinsic Immunity

#### ICP0 Deploys Multiple Strategies to Antagonize PML-NB Restriction Factors

PML-NB constituent proteins (e.g., PML, SP100, Daxx, ATRX, MORC3, and PIAS1) failed to restrict WT HSV-1 infection due to the presence of ICP0 (Everett et al., 2006, 2008a; Lukashchuk and Everett, 2010; Brown et al., 2016; Sloan et al., 2016). During the initial stages of infection, ICP0 localizes to PML-NBs prior to mediating their disruption. ICP0 employs multiple mechanisms to do so (Boutell and Everett, 2013; Gu et al., 2013). It shares many features with SUMO-targeted ubiquitin ligases (STUbL), which are a family of enzymes that contain SIMs that mediate interactions with SUMO-modified proteins (Boutell et al., 2011). Seven SIM-like sequences (SLS1–7) have been identified within the ICP0 open reading frame. During the initial stages of infection, ICP0 localizes to SUMO1 and SUMO2/3 conjugates and preferentially targets them for proteasomal degradation in a RING finger-dependent manner. Proteomics analysis has identified 124 proteins that showed reductions (≥threefold) in the levels of their SUMO-modified forms during HSV-1 infection (Sloan et al., 2015). SUMO-modified PML and SP100 are well-known target substrates for ICP0 (Chelbi-Alix and de The, 1999; Boutell et al., 2003, 2011). SLS4 has been shown to be necessary for ICP0 interaction with SUMO2/3 and targeting of SUMO-modified PML for degradation. Moreover, multiple mutations within ICP0 SLSs (SLS4–7) rescued SUMO-conjugated proteins from degradation and reduced the ability of ICP0 to rescue the plaque formation ability of ΔICP0 HSV-1 (Boutell et al., 2011). However, ICP0 also employs a SUMO-independent mechanism for PML targeting (Cuchet-Lourenco et al., 2012). It directly interacts with PML isoform I (PML.I) and induces its degradation. This process occurs independently of the PML.I SIM, and it instead depends on the PML.I-specific exon 9 in the N-terminal half of ICP0 (Cuchet-Lourenco et al., 2012).

Recently, MORC3 was identified as a target substrate for ICP0 (Sloan et al., 2015, 2016). During WT HSV-1 infection, a high degree of colocalization between ICP0 and MORC3 was observed during the initial stages of infection prior to the degradation of SUMO-modified and unmodified MORC3. This process occurred in an ICP0 RING finger-dependent manner, but independently of SLS4–7 (Sloan et al., 2016). Whether ICP0 directly interacts with MORC3 remains to be determined.

Other PML-NB restriction factors (e.g., Daxx, ATRX, and PIAS1) are not degraded during WT HSV-1 infection (Lukashchuk and Everett, 2010; Brown et al., 2016). In fact, ICP0 failed to directly interact with these proteins, as demonstrated by co-immunoprecipitation assays (Lukashchuk and Everett, 2010; Brown et al., 2016). However, the presence of ICP0 blocks their recruitment to infecting viral genomes and efficiently counteracts their repressive antiviral activity, possibly by degrading other PML-NB restriction factors such as PML and MORC3, leading to PML-NB disruption (Lukashchuk and Everett, 2010; Brown et al., 2016; Sloan et al., 2016; Alandijany et al., 2018).

Thus, HSV-1 can efficiently counteract PML-NB-mediated silencing of the viral genomes. The viral E3 ubiquitin ligase ICP0 employs SUMO-dependent and -independent targeting mechanisms to mediate the degradation and dispersal of host restriction factors away from viral genomes to promote the onset of lytic infection.

#### ICP0 Impairs the Intrinsic Restriction Mediated by DDR Proteins

During WT HSV-1 infection, the formation of irradiation-induced foci (IRIF) and the accumulation of DNA repair proteins at IRIF are disrupted by the viral E3 ubiquitin ligase ICP0 (Lilley et al., 2010, 2011). Indeed, ICP0 induces the degradation of the RNF8 and RNF168 ubiquitin ligases required for the accumulation of DNA repair proteins in an ICP0 RING finger- and cellular proteasome-dependent manner. In infected cells, cellular CK1 kinase phosphorylates ICP0, thereby creating a “mimic” of a cellular phosphosite, which promotes ICP0 interaction with RNF8, eventually leading to the degradation of RNF8 (Chaurushiya et al., 2012). Degradation of these cellular ubiquitin ligases (RNF8 and RNF168) leads to a substantial loss of ubiquitinated H2A and H2AX, which impairs DNA repair protein recruitment and IRIF formation. Therefore, the plaque formation of WT virus, unlike ΔICP0 HSV-1 and ICP0 RING finger mutants, was not affected by RNF8 ectopic expression, which highlights the key role of ICP0 in evading intrinsic repression mediated by DNA repair proteins (Lilley et al., 2010, 2011; Chaurushiya et al., 2012).

#### Epigenetic Repression of Viral Genome Is Also Counteracted by ICP0

In addition to VP16, the viral protein ICP0 is key in reversing the association of epigenetic repressors with viral genomes (Herrera and Triezenberg, 2004; Lee et al., 2016). VP16 initially promotes the removal of histone H3 from IE promoters and enhances the recruitment of transactivation factors (e.g., HCF-1 and Oct-1) to stimulate viral gene expression, including ICP0 expression (Herrera and Triezenberg, 2004). Thereafter, ICP0 mediates heterochromatin (H3K9me3) removal on viral E gene promoters (e.g., ICP8), and induces the degradation of free histones (H2B) to minimize their availability to bind the vDNA (Lee et al., 2016). Furthermore, ICP0 disrupts the REST/CoREST/HDAC nuclear repressor complex in order to enhance the transition from IE to E protein expression. ICP0 binds to CoREST, translocates CoREST and HDAC to the cytoplasm, and promotes the dissociation of HDAC (Gu et al., 2005; Gu and Roizman, 2007, 2009). As the infection progresses, encapsidation of viral genomes further contributes to the removal of core histones and makes the vDNA inaccessible to epigenetic repressors.

### ICP0-Mediated Impairment of Host Innate Immunity

HSV-1 efficiently counteracts many aspects of host innate immunity, including evasion of PRR recognition (e.g., IFI16 degradation and inhibition of the cGAS-mediated signaling pathway), modulation or blocking of immune signaling cascades (e.g., TRIM29-mediated degradation of STING and disruption of TBK1-IRF3 interaction), and interference with effector protein functions (e.g., degradation of ISG mRNAs) (Verpooten et al., 2009; Zenner et al., 2013; Shen et al., 2014; Su et al., 2015; Orzalli et al., 2016; Xing et al., 2017; Xu et al., 2017; Tognarelli et al., 2019). Many HSV-1 proteins have been found to be involved in the innate immune evasion. ICP0 plays a central role in this process (Lanfranca et al., 2014; Orzalli and Knipe, 2014). In fact, the presence of ICP0 has been shown to inhibit both IFN-induced and viral-induced ISG expression (Harle et al., 2002; Mossman and Smiley, 2002; Melroe et al., 2004; Paladino et al., 2010). Correspondingly, robust induction of ISGs was only observed during infection with HSV-1 mutants that failed to express ICP0, and these mutants were hypersensitive to IFN pretreatment compared to WT virus (Mossman et al., 2000; Eidson et al., 2002; Harle et al., 2002; Everett and Orr, 2009).

Multiple mechanisms for ICP0-mediated inhibition of innate immunity have been proposed (Lanfranca et al., 2014). As discussed above, ICP0 induces PML degradation at the early stage of infection to release the viral genomes entrapped within PML-NBs. By doing so, ICP0 also counteracts host innate immunity because the presence of PML is important for efficient induction of ISG expression (Alandijany et al., 2018; McFarlane et al., 2019). ICP0 has also been shown to induce the degradation of the vDNA sensor IFI16 in a RING-dependent manner (Orzalli et al., 2012; Cuchet-Lourenco et al., 2013; Johnson et al., 2013). However, IFI16 degradation occurred at a slower kinetic rate in comparison to PML degradation (Cuchet-Lourenco et al., 2013). Also, it remains controversial whether ICP0 is directly required and sufficient for IFI16 degradation (Cuchet-Lourenco et al., 2013; Orzalli et al., 2016).

Impairment of IRF3 function is another strategy employed by ICP0 to counteract innate immunity. Nuclear ICP0 binds to IRF3 and its binding partner CBP, leading to the formation of the ICP0/IRF3/CBP nuclear complex. This interaction sequesters IRF3 away from the host chromatin and prevents ISG expression (Melroe et al., 2004, 2007). Although ICP0 expression is predominantly nuclear, ICP0 translocates to the cytoplasm as the infection progresses (Lopez et al., 2001). A study suggested that cytoplasmic ICP0 promotes viral replication by blocking the activation of IRF3 and preventing the induction of innate immunity (Taylor et al., 2014).

ICP0 does not only impair the host IFN response, but it also interferes with the NF-κB signaling pathway via several mechanisms, including degradation of TLR2 and p50, and blocking of p65 nuclear import (van Lint et al., 2010; Zhang et al., 2013). Collectively, these studies demonstrate that ICP0 impedes the induction of host innate immunity during HSV-1 infection in addition to its key role in antagonizing intrinsic antiviral restriction.

## Conclusion

Intrinsic and innate immunity are two distinct arms of host intracellular antiviral responses to HSV-1 infection. The differences between these two arms include the induction requirements, the nature of the effector proteins, and the antiviral effects on viral replication. The intrinsic antiviral response is mediated by pre-existing host cell restriction factors that immediately recognize vDNA upon nuclear entry and directly repress the onset of viral replication by inducing viral genome silencing. On the other hand, the induction of the innate immune response is triggered following the escape of viral genomes from intrinsic silencing and the initiation of viral gene expression and DNA replication. It is mediated by PRRs, which recognize viral components and replication intermediates/products, leading to IFN production and ISG expression. The induction of ISG products with antiviral properties in the infected and neighboring uninfected cells inhibits viral propagation and limits the spread of infection. The presence of the viral countermeasure ICP0 initially antagonizes the intrinsic repression of viral genomes and it subsequently impairs innate immunity induction. This sequential regulation of intracellular immunity remained unidentified for many years, and it would not have been possible to characterize this regulation process without studies utilizing physiologically relevant low MOI conditions. Click chemistry-mediated detection of vDNA prelabeled with pyrimidine deoxynucleotide was key in advancing our understanding of these temporally regulated very early events of the cellular antiviral response. These findings on HSV-1 infection likely apply to other viral infections and are worthy of further investigation.

## Author Contributions

TA: conceptualization, writing the original draft, reviewing, and editing.

## Conflict of Interest

The authors declare that the research was conducted in the absence of any commercial or financial relationships that could be construed as a potential conflict of interest.
